# Empirical Evaluation of Visual Fatigue from Display Alignment Errors Using Cerebral Hemodynamic Responses

**DOI:** 10.1155/2013/521579

**Published:** 2013-12-24

**Authors:** Hanniebey D. Wiyor, Celestine A. Ntuen

**Affiliations:** Department of Industrial & Systems Engineering, North Carolina A&T State University, 1601 East Market Street, 406 McNair Hall, Greensboro, NC 27411, USA

## Abstract

The purpose of this study was to investigate the effect of stereoscopic display alignment errors on visual fatigue and prefrontal cortical tissue hemodynamic responses. We collected hemodynamic data and perceptual ratings of visual fatigue while participants performed visual display tasks on 8 ft × 6 ft NEC LT silver screen with NEC LT 245 DLP projectors. There was statistical significant difference between subjective measures of visual fatigue before air traffic control task (BATC) and after air traffic control task (ATC 3), (*P* < 0.05). Statistical significance was observed between left dorsolateral prefrontal cortex oxygenated hemoglobin (*l* DLPFC-HbO_2_), left dorsolateral prefrontal cortex deoxygenated hemoglobin (*l* DLPFC-Hbb), and right dorsolateral prefrontal cortex deoxygenated hemoglobin (*r* DLPFC-Hbb) on stereoscopic alignment errors (*P* < 0.05). Thus, cortical tissue oxygenation requirement in the left hemisphere indicates that the effect of visual fatigue is more pronounced in the left dorsolateral prefrontal cortex.

## 1. Introduction

The visual system is a well-developed sensory organ that involves effortless complex interaction between the visual organ and the brain. It involves physiological and a series of hierarchical neural processes from the photoreceptors, retinal ganglion cells, the lateral geniculate nuclei (LGN) of the thalamus, and the visual cortices [1]. The American Optometric Association [2] identified that prolonged use of the visual system can result in inefficient visual processing functions generally called visual fatigue. In line with that, the American Association of Ophthalmologist (AAO) diagnosed visual fatigue as an ophthalmological disease called asthenopia with the medical diagnostic code of ICD-9, 368.13 with the subclassifications shown in Table 1 [3, 4]. The last two digits of the medical code indicate the seriousness of the ophthalmological disease. The larger the last two digits, the more severe the symptoms of the disease.

Visual fatigue commonly arises when people view stereoscopic motion images [5]. In a stereoscopic image, the viewer watches two images corresponding to the right and left eyes with convergence eye movement. Based on individual characteristics and instrumentation issues, conflicts can occur between convergence or divergence eye movements and the accommodation function thus causing visual fatigue [6]. One of the major problems with stereoscopic displays is that a stereoscopic display produces a mismatch between the focus (accommodation distance) and the fixation (convergence distance) of human eyes. Thus, stereoscopic images may present different depth information to the accommodation and convergence functions. Stereoscopic displays require that the viewer converges and accommodates at different depth planes, thus creating a conflict between the two visual mechanisms [7]. This problem is common in 3D display systems such as Head Mounted Displays (HMDs) and displays that make use of a lenticular lens. Imbalanced visual information may be one of the reasons for the visual load and eventually can cause the visual fatigue associated with 3D images [8]. Further, Inoue and Ohzu [8] noted that the common cause of visual fatigue in a stereoscopic display is the fact that 3D spaces generated by stereoscopic images are artificial spaces which are different from real spaces. As a result, the perceived images through 3D glasses do not really exist. Okada et al. [9] and Fukushima et al. [10] showed that improvements of image quality, when a stereoscopic system is used, increase the discrepancy of accommodation and convergence and may cause increased visual fatigue. For example, in one of their experiments, Kuze and Ukai [11] had participants playing a video game using a stereoscopic HMD or monoscopic TV display and found significantly greater increases in subjective ratings of general discomfort, nausea, and headache after using the HMD compared to the increases observed with a conventional TV display.

An issue not often discussed in stereoscopic display research is alignment and calibration errors. Three types of alignment errors in optical systems are defined by Self [12]. Vertical alignment error is an upward or downward tilt in the optical axis of one image resulting in differences in vertical position of an image. Rotational alignment error is a tilt in one of the images with the degree of alignment error increasing from the center. Lastly, magnification difference is an error due to the size difference between the left and right stereo pairs from the field of view. Alignment errors have two main sources [13]. Firstly, these are errors in optics due to a shift in the image placement of the left and right stereo pairs. These optical errors are due to either a shift in rotation, a difference in magnification, or a difference in reduction. Second, filtration errors in photometry caused by the difference in luminance, color, contrast, chromatic aberration, or crosstalk (images switching back and forth between the eyes). The existing past studies have not explicitly addressed the issues of image alignment errors on the cerebral cortical responses and visual fatigue severity ratings. This study investigates the effect of prolonged use of stereoscopic display on visual fatigue and dorsolateral prefrontal cortex hemodynamic responses during visual display tasks.

### 1.1. Contribution of This Paper

The intellectual contribution of this paper can be realized in ophthalmological disease etiology, optometry, vision science, and 3D devices and systems. Specifically, the neuroimaging of the brain in relation to visual fatigue can yield rich neurophysiologic data to objectively and clinically classify and diagnose onsets of visual fatigue.

## 2. Methods

### 2.1. Participants

This study included 21 males and 3 females recruited from North Carolina Agricultural and Technical State University community. Their ages ranged from 18 to 40 years. All participants self-reported no history of ophthalmological diseases and optometric corrections. Participants' visual acuity and color blindness score were limited to 20/40 and 16 out of 22 points, respectively, and they were compensated for their participation. The University's Institutional Review Board approved all aspects of the research.

### 2.2. Instrumentation and Data Acquisition

The apparatus for the study were a 8 ft × 6 ft. NEC LT silver screen, NEC LT 245 DLP projectors, NEC LT 245 polaroid glasses, a 22′′ 1.58 GHz 0.99 GB RAM Gateway DCDi Desktop, a Snellen Chart, and a Simulator Sickness Questionnaire (SSQ), a neurophysiological assessments, and an ophthalmological questionnaire. Several software systems were used for the study. These were Air Traffic Control Interface Model (ATCIM), Cognitive Optical Brain Imaging Studio (COBI) provided by fNIRS Devices LLC (2010) for the fNIRS. Raw materials used included 70% Isopropyl Alcohol Swabs, Cotton Squares, and AstroMed bandages.

#### 2.2.1. Simulator Sickness Questionnaire (SSQ)

The SSQ as proposed by Kennedy et al. [14] is comprised of four parts: nausea rating, oculomotor fatigue rating, disorientation rating, and a total score rating. It consists of thirty-two symptoms and for each symptom, a higher rating indicates that a participant experienced more symptoms. The symptoms are rated as None = 0; Slight = 1; Moderate = 2; and Severe = 3.

#### 2.2.2. Functional Near Infrared Spectroscopy (fNIRS)

fNIRS is a brain imaging device for exploring brain responses to a variety of stimulated cognitive and physical tasks in the field of cognitive neuroscience. This device consists of an infrared light source with a wavelength range of 650–850 nm into the head and a detector that receives light after it has interacted with the tissue [15]. The emitted photons after interacting with head tissues undergo two types of interaction, namely, absorption (loss of energy to the medium) and scattering [16–18]. The spatial resolution in fNIRS is limited to approximately 5 mm. Researchers have shown that by placing the probes on a Participant's forehead, fNIRS provides accurate measures of activities within the frontal lobe of the brain which are responsible for many high order cognitive functions, such as memory and problem solving [15]. The light passing through the frontal tissues is sufficiently low to allow for tissue imaging at depths up to 20–30 mm. Deoxygenated (Hbb) and oxygenated (HbO_2_) hemoglobin are the main absorbers of near infrared light in tissues, and they provide relevant markers of hemodynamic and metabolic changes associated with neural activity in the brain. The levels of Hbb and HbO_2_ measured are relative to the baseline only. It is not possible to arrive at absolute values of concentration using fNIRS imaging on living samples. Once the Hbb and HbO_2_ levels are computed, they are used to calculate the levels of oxygenation (in *μ*M) and values that may be approximately treated as percent changes in blood volume.

#### 2.2.3. Air Traffic Control Interface Model (ATCIM)

**Figure 1:**
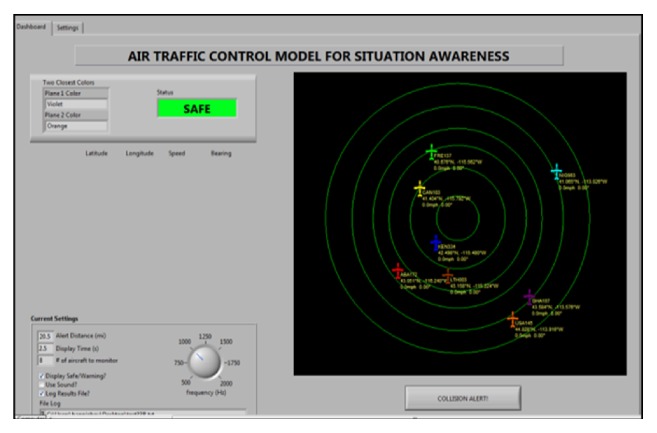
ATCM interface designed with LabView 9.1 software provided National Instrument Wiyor [19].

The task stimulus is an Air Traffic Control Interface Model (ATCIM) developed by Wiyor [19] and designed with LabView 8.1. provided by National Instrument [20]. The simulated testbed provides a situation awareness (SA) environment similar to an ATC task domain. The use of ATCIM as an ATC simulator is specifically designed to allow for close experimenter control over display features and task performance, while providing a moderate degree of realism. In the ATCIM domain, an operator perceives and responds to aircraft conflict separation tasks which occur when two or more aircrafts are in self-separation violation. The separation violation is set to a loss of separation of 5 NM laterally or 500 ft. vertically or both conditions can occur simultaneously [21]. The ATCIM is shown in Figure 1.

The ATCIM system consists of two main interfaces, namely, the control setting interface and the dashboard interface. The control setting interface allows the user to select inputs for the simulation. These are (a) two closest aircraft color identifications; (b) a color status bar in which two mutually exclusive red and green colors appear with green for safe mode and red for crash mode; (c) an alert distance control that allows the user to preset the minimum safe distance between two planes; (d) a display time control which allows for the user to input the maximum time allowed for a participant to perceive and respond to a collision alert; (e) a number of aircraft to monitor which allows the user to preselect the number of aircraft for the experiment; (f) a collision alert used by the participants to respond before collision time; and (g) an aircraft information-radii mode in which the plane path, latitude, longitude, speed, and bearing are displayed on the radii and move with the aircraft.

The dashboard interface has an output section for data collection during the simulation. This consists of a 5 NM (nautical miles) radius sector in which the number of aircrafts is selected to be monitored by the participants. Based on the predetermined mode selected from the setting section, the required output will be shown in the dashboard section at each simulation run. Each aircraft is characterized by traffic and conflict information. Within the ATCIM, the minimum safe altitude warning distance is fixed to a default value.

### 2.3. Experimental Procedures and Protocol

The experiment was conducted as completely randomized within participant design. The experimental treatments are three stereoscopic alignment errors and each treatment was replicated twice. It should be noted that a particular treatment replication is completed before moving to the next treatment. For example, if the randomization order is vertical shift, magnification difference, and rotational error, the vertical shift treatment will be replicated twice and each replication will last for 10 minutes, hence 20 minutes for vertical shift as ATC1, followed by magnification difference; two replications as ATC2; and lastly, the rotational error with two replications as ATC3.  Table 2 contains the information on the independent variable, levels, and measurement units for the experiment. The experimental treatments were characterized by cognitive loading and task difficulty. The cognitive loading was the number of aircraft on the radar with separation conflicts. The number of aircraft ran from low being 3 aircraft and high being 9 aircraft.

Task difficulty was characterized by separation conflicts labeled as vertical, horizontal, combined horizontal and vertical, and none, respectively. A high maneuver (ATC3) had a maximum number of nine aircraft (high cognitive load) with either combined horizontal and vertical or none for separation conflicts, and a low maneuver (ATC1) had 2-3 aircraft conflicts in the airspace.

The independent variable was the stereoscopic alignment errors observed at three levels, namely, vertical shift, rotational error, and magnification difference. For the vertical shift, the focused images on the left and right retina were displaced by 0.5′; the rotational error had the images displaced from each other in the left and right retina by 0.25′; and for magnification difference, an error was caused by 6.25% enlargement in one image. The two response measures in the study were cerebral hemodynamic response and visual fatigue ratings. The cerebral hemodynamic responses were continuous variables measured with fNIRS and visual fatigue symptoms were subjective ratings measured by SSQ.

Each participant was given an informed consent to read and sign. Verbal explanations were provided to clarify concepts and terminologies. The neurophysiological assessment and ophthalmological assessments survey questionnaires were administered. The ophthalmological survey was used to ensure that the participants under 40 years had no optometric and/or ophthalmological symptoms. Available studies have shown that after age 40, most people experience the onset of ocular-motor problems, such as accommodations and convergence [22, 23]. The National Eye Institute [24] estimates that, currently, more than 38 million Americans age 40 and older experience blindness, low vision, or an age-related eye disease, such as age-related macular degeneration (AMD), glaucoma, diabetic retinopathy, or cataracts. This is expected to grow to more than 50 million by year 2020 [24]. In addition, a subjective neurological questionnaire was administered to participants for life styles and/or accidental damage that might affect the brain electrophysiological data.

**Figure 2:**
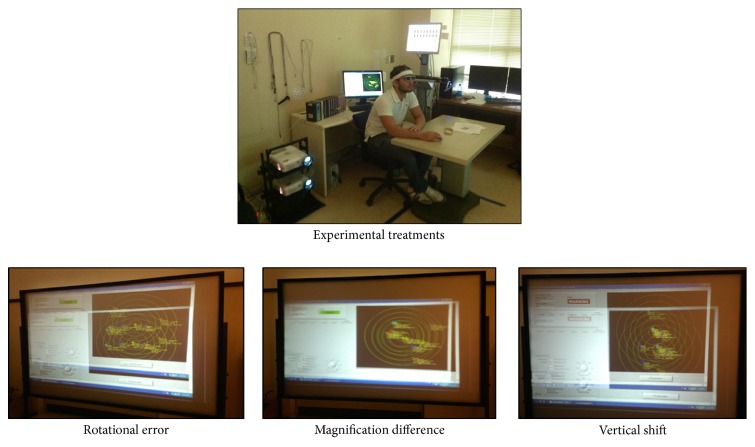
Participant performing experimental tasks with various alignment error conditions.

Each participant was assigned an alphanumerical identification number instead of using their real names; the same identification number was also assigned to a particular randomized experimental treatment in accordance with the IRB protocol. The participants' foreheads were cleaned with 70% Isopropyl Alcohol Swab and air-dried for two minutes. The fNIRS sensor pad was placed on the participant's forehead and held in place by a head band. Participants were introduced and welcomed to the experimental station. The participants were then seated about 120 inches in front of the 8 ft × 6 ft silver screen as shown in Figure 2. They were then given SSQ consisting of 32 symptoms to rate their perception of visual fatigue as none (0), slight (1), moderate (2), and severe (3).

The participants proceeded to perform the ATC task presented in alignment error labeled as ATC1. After ATC1, participants rated the SSQ symptoms. Subsequently, they proceeded to perform the same ATC task but with a different alignment error labeled as ATC2. The procedure was followed by SSQ ratings. Lastly, the participants performed the ATC task (labeled ATC3) under alignment errors, followed by SSQ ratings. It should be noted that throughout the experiment participants were continuously asked if they were willing to continue the experiment. The tasks involve continuous monitoring where the participants visually scanned the display to detect any two or more self-separation violations. The participants responded by pressing a “CLICK BUTTON” located at the interface of the ATCIM if a violation was detected. However, if they detected no self-separation violations, no response was issued and after 20 seconds, the display refreshed itself to a new display.

The self-separation violation was set to a loss of separation of 5 nmi laterally or 500 ft. vertically [25]. After the completion of each session, participants were asked to rate their perception of visual fatigue. The participants were required to perform the experimental tasks as quickly and as accurately as possible. The average time for each experiment was 1 hr. 45 mins, inclusive of IRB briefing, visual acuity test, conducting the ophthalmological and neurological survey questionnaires, performing the experiments, and cleaning the participants and debriefing them.

### 2.4. Data Collection and Signal Preprocessing

Four SSQ instruments were administered per participant after each experimental treatment labeled as BATC, ATC1, ATC2, and ATC3. For analyses, the files were generated by conducting frequency tallying each participant's responses for each experimental treatment. After obtaining the total number of responses per symptom per treatment, the total adjusted rating for each symptom was calculated as

(1) Adjusted ratings for symptoms=∑n=03WnXn∑n=03Xn,

where *n* = 0, 1, 2, and 3 are the index counts for None, Slight, Moderate, and Severe SSQ ratings of visual fatigue for a particular symptom, *W*_*n*_ is the total rating for *X*_*n*_, and *X*_*n*_ has the values

(2) Xn={X0=0;NoneX1=1;SlightX2=2;ModerateX3=3;Severe.

The total weighted rating across all 32 symptoms per treatment was calculated as shown in:

(3) ({(∑n=03WnXn∑n=03Xn)}S1⋯ +{(∑n=03WnXn∑n=03Xn)}S32)×(N=24)−1,

where *N* is total number of participants; *S*1, *S*2,…, *S*32 represent the types of symptoms rated.

An fNIRS sensor pad was placed on the participant's forehead specifically at the dorsolateral prefrontal cortex and held in place by a head band. The hemodynamic data was recorded using fNIRS (Model: 1000, fNIR Devices LLC, MD, USA) at a sampling rate of 100 kHz, a frame rate of 500 ms, and samples per voxels of 25. The data acquisition settings included an LED drive current of 10 mA, an Analog-to-Digital (A/D) gain of 1 mA, and an initial gain drift of 1 with a balance of 4000.

There were two main hemodynamic responses measured by fNIRS. These responses were changes in oxyhemoglobin concentration Δ*C*_HbO_2__ (*t*) and deoxyhemoglobin concentration Δ*C*_Hbb_ (*t*) from the 16 voxels. The recordings cover the area of dorsolateral prefrontal cortex (DLPFC). The DLPFC roughly corresponds to Broadman areas 9 and 46, and it covers portions of the middle and inferior frontal gyri [26]. These regions of interest for task execution in the prefrontal area had been identified based on a previous fNIRS study by Izzetoglu et al. [15] and based on a meta-analysis of other neuroimaging studies performed by Cabeza and Nyberg [27]. To reduce the data dimensionality, participant averages were computed using Microsoft Excel 2009 for both oxyhemoglobin and deoxyhemoglobin concentration for the 16 voxels as HbO_2 (mean)_ and Hbb_(mean)_, respectively. As indicated by fNIR Devices LLC [28], the voxels 1 to 8 correspond to the left (*l*) DLPFC while voxels 9 to 16 correspond to right (*r*) DLPFC. For each of the two traditional hemodynamic variables, the mean value (*H*_mean_), the maximum value (*H*_max_), and standard deviation value (*H*_stdev_) were computed.

## 3. Data Analyses and Results 

The data preprocessing consisted of two steps. First, each of the dependent variables was preprocessed for outliers, missing values, or nonresponses. For the missing values, they were replaced by the computed average of the values [29]. The second approach involved applying the appropriate software and technique to each dependent variable to make it suitable for statistical analysis. A model adequacy check was performed to test for the three ANOVA assumptions of normality, independence, and homogeneity of variance. If the original data violated any of the assumptions, an appropriate transformation was applied to the data until all the assumptions were met. The model adequacy was analyzed with statistical analysis software (SAS) [30]. The descriptive statistics for hemodynamic responses is summarized as shown in Table 3.

### 3.1. Psychophysiological Ratings of Visual Fatigue

Cronbach's alpha reliability test was applied to the SSQ responses using SAS [30]. The Cronbach was evaluated based on participants overall SSQ scores for each ATC task session (alignment errors). Thus, the dataset consisted of twenty-four observations and four attributes representing BATC, ATC1, ATC2, and ATC3. A Cronbach alpha 0.961 was obtained. This shows stable responses considering 0.7 as the cutoff for acceptable response [31–34].

**Figure 3:**
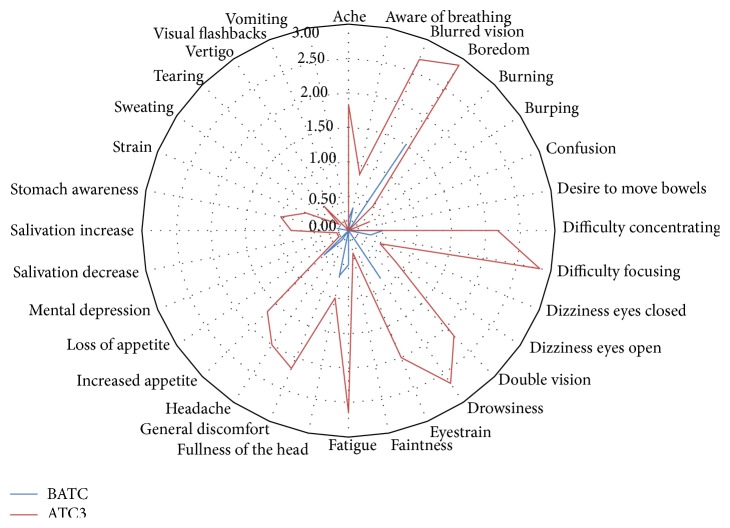
A radial plot of weighted mean SSQ rated symptoms on BATC and ATC3 task sessions.

A paired *t*-test statistical technique (*α* = 0.05) was used and the data was analyzed by SAS [30]. At 5% significance level, there was enough evidence to conclude that a prolonged use of stereoscopic display was likely to induce visual fatigue as there was a significant difference between the SSQ responses between before air traffic control task (BATC) and after air traffic control task (ATC 3), *t* (23) = −15.27, *P* < 0.05. An exploratory analysis revealed that twenty-one of the thirty-two symptoms were pronounced after ATC3. This is shown in Figure 3. These observed significant symptoms include ache, blurred vision, boredom, difficult concentrating, difficult focusing, dizziness eye open, dizziness eye closed, double vision, drowsiness, eyestrain, faintness, fatigue, fullness of the head, general discomfort, headache, loss of appetite, increased appetite, mental depression, salivation increase, salivation decrease, and tearing.

Further, one-way ANOVA analysis (*α* = 0.05) was conducted to determine the effect stereoscopic alignment errors on SSQ ratings. At 5% level of significance, there was enough evidence to conclude that at least one of the stereoscopic alignment errors was different, the one-way ANOVA results, *F* (2, 69) = 35.38, *P* < 0.05. A Tukey post ad hoc analysis revealed that there was a significant difference between magnification difference and vertical shift (*P* < 0.05) and magnification difference and rotational error (*P* < 0.05). There was no significant difference between vertical shift and rotational error.

### 3.2. Tissue Hemodynamic Responses

A one-way MANOVA statistical technique (*α* = 0.05) was used to analyze the effect of display alignment errors on cerebral tissue hemodynamic responses with SAS. For any significance, a Tukey post ad hoc analysis was conducted to reveal the significant difference. For the hemodynamic responses, the Hbb variable, *W* = 0.406, *P* < 0.05, and HbO_2_ variable *W* = 0.476, *P* < 0.05. The normality tests were violated. As a result of significant violations of the model adequacy checks by the hemodynamic data, the data underwent some transformations. Various transformation approaches were applied to the hemodynamic response data and further analyzed for model adequacy check. Eventually, the box-cox transformation yielded the best results. The dataset was transformed by a power of 1/3 (that is *X* ^1/3^). After the transformation, Shapiro-Wilks test showed that *W* = 0.04, *P* = 0.65; for HbO_2_ variable, *W* = 0.015, *P* = 0.548. Levene's test for homogeneity of variance for Hbb and HbO_2_ variables, analyzed with ANOVA of squared deviations from group mean, had significant results for Hbb, *F* (5, 272) = 1.23, *P* = 0.541, and HbO_2_, *F* (5, 272) = 0.74, *P* = 0.75.

There was a low to moderate correlation between the hemodynamic variables. All the variables were positively correlated with the exception of (*l*) DLPFC-Hbb and (*r*) DLPFC-HbO_2_, which had negative correlation between them as shown in Table 4. This means that as an activity increases in the left dorsolateral prefrontal cortex, the less oxygen is demanded in the right dorsolateral prefrontal cortex.

From Table 4, *r* DLPFC-Hbb was positively correlated with *r* DLPFC-HbO_2_. This means that an increase in activity in the right dorsolateral prefrontal cortex requires an increase in oxygen demand. We noted that *r* DLPFC-HbO_2_ was also positively correlated with *l* DLPFC-HbO_2_. Thus, any oxygen requirement in the right dorsolateral prefrontal cortex results in equivalent increase in demand in the left dorsolateral prefrontal cortex (a positive correlation of 0.44). Further, *r* DLPFC-Hbb has a positive correlation of 0.55 with *l* DLPFC-Hbb, meaning that performance of visual activities is concurrent in both right and left dorsolateral prefrontal cortex.

The one-way MANOVA results showed Wilk's Lambda = 0.924, *F* (8, 132) = 0.66, *P* < 0.05. Thus, at 5% level of significance, there was enough evidence to conclude that the transformed hemodynamic response as a composite score was significant. Further, at 5% level of significance, there was enough evidence to conclude that at least one of the stereoscopic alignment errors was different: for *l* DLPFC-Hbb transformed dataset, the ANOVA result, *F* (2, 69) = 0.10, *P* < 0.05, for *l* DLPFC-HbO_2_ transformed dataset, the ANOVA result, *F* (2, 69) = 0.15, *P* < 0.05, and *r* DLPFC-Hbb transformed dataset, the ANOVA result, *F* (2, 69) = 1.09, *P* < 0.05. Tukey analyses for *l* DLPFC-Hbb, *l* DLPFC-HbO_2_, and *r* DLPFC-Hbb results are shown in Table 5.

## 4. Discussion

**Figure 4:**
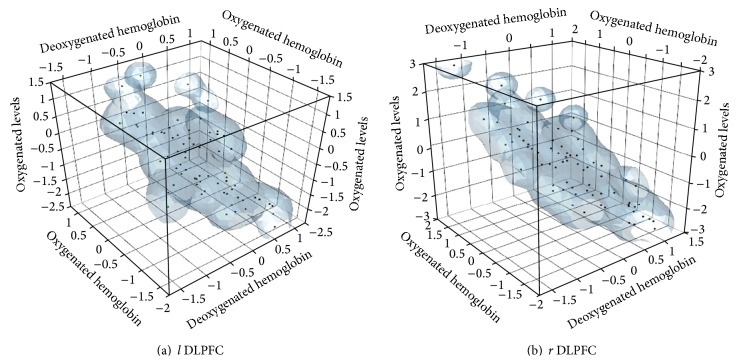
Temporal patterns for hemodynamic responses for the dorsolateral prefrontal cortex for all the alignment errors during ATC tasks.

It was possible to elicit tissue hemodynamic response and Simulator Sickness Questionnaire (SSQ) ratings based on visual task in stereoscopic alignment errors. The stereoscopic alignment errors had great impact on the SSQ responses. From the second to the third experimental sessions, the participants' perceptual ratings of visual fatigue increased from slight to moderate or moderate to severe. The left dorsolateral prefrontal cortex was affected more than the right dorsolateral prefrontal cortex. The oxygenated hemoglobin and deoxygenated hemoglobin in the left dorsolateral prefrontal cortex were significantly affected by the stereoscopic display alignment error. While in the right dorsolateral prefrontal cortex, the stereoscopic display alignment error had an impact on only the deoxygenated hemoglobin. As observed by Buxton et al. [35], responses to stimuli changes result in increase or decrease of regional cerebral blood flow (rCBF) to this localized brain region. It increases with the increase in demand for decision making processes. The cortical tissue oxygenation requirement in the left hemisphere indicates that the effect of visual fatigue is more pronounced in the left dorsolateral prefrontal cortex. Using the oxygenated hemoglobin, deoxygenated hemoglobin, blood volume, and oxygenation levels, a 3D scatter plot for neuroimaging data comprising deoxygenated hemoglobin, oxygenated hemoglobin, and oxygenation level was plotted as shown in Figure 4. Increases in the oxygenated hemoglobin resulted in a corresponding increase in the oxygenated levels. This was depicted by the upward movements toward the vertex of the oxygenation level and oxygenated hemoglobin variables.

The same pattern was depicted in the right dorsolateral prefrontal cortex. However, for the right dorsolateral prefrontal cortex, the distribution was narrower than the left dorsolateral prefrontal cortex. The ATC tasks induced more cognitive processes in the left dorsolateral prefrontal cortex than the right dorsolateral prefrontal cortex. As a result of cognitive load, more energy was required by the brain with a corresponding oxygen requirement. According to Hansen [36], the concentration of oxygen in the brain is about 0.1 *μ*mol g^−1^, of which 90% is in oxy-Hbb in brain capillaries. This concentration can support the normal oxygen consumption of about 3.5 *μ*mol g^−1^ min^−1^ for two seconds [37]. For this reason, any increase in neural activity in the brain is followed by the rise in regional cerebral blood flow (CBF) [35]. Oxygen is transported to neural tissue via oxygenated hemoglobin (oxy-Hbb) in the blood. The demand for glucose and oxygen by neuronal tissues in a particular brain region may vary due to particular processing requirements at a particular time.

Increases in hemoglobin and oxygenation levels in left dorsolateral prefrontal cortex mean that the left side of the brain is more engaged with visiomotor cognitive processes than the right side of the brain. The continual engagement of the left brain may lead to mental fatigue. The post ad hoc analyses revealed that there were significant differences between magnification error and rotational error and magnification error and vertical shift error. However, there was no significant difference between rotational error and vertical shift error. For the right hemisphere of the dorsolateral prefrontal cortex, oxygenated hemoglobin, blood volume level, and oxygenation levels were significant. Since magnification difference errors were significant, we conducted a further analysis to identify likely points of visual fatigue. The hemodynamic responses revealed that significant differences exist between left and right dorsolateral prefrontal in the alignment errors in visual attention tasks, *F* (1, 47) = 0.034, *P* < 0.05. Generally, oxygenation levels were increased in both left and right dorsolateral prefrontal; however, it was more pronounced in the left dorsolateral prefrontal. In the left dorsolateral prefrontal, the increased oxygenation levels resulted from the corresponding increased oxygenated hemoglobin and blood volume. Contrasting to the left dorsolateral prefrontal, oxygenated levels increased without any blood flow to that region. More interestingly, the pronouncements in left dorsolateral prefrontal can be traced to the fact that all participants were right-handed.

## 5. Conclusions

Regrettably, it is well documented that the introduction of three-dimensional displays such 3D TV, High-Definition Multimedia Interface (HDMI) desktop, Head-Mounted Display, Helmet-Mounted Display, and 3D glass into consumer markets has resulted in the increase of oculist visits for visual complaints [38, 39]. From the empirical findings, stereoscopic display alignment errors can induce visual fatigue with accompanying underpinning physiological effects associated with visual task performance. Visual fatigue is likely to increase the hemodynamic response in the left dorsolateral prefrontal cortex of the brain and delta band waves can be used to predict cognitive fatigue across all the occipital cerebral cortex. To improve 3D or stereoscopic display systems for minimum visual fatigue to the users, the optical axes system in display devices should be designed with minimum magnification difference and rotational error, as these errors were observed to be most prevalent. 3D display designs with minimum magnification difference and rotational error are more likely not only to reduce visual fatigue but also to increase performances and satisfaction of the users such as aviators and flight pilots.

## Figures and Tables

**Table 1:** Ophthalmological classification of visual fatigue (asthenopia) with their respective symptoms.

Ophthalmological medical code	Ophthalmological symptoms
ICD-9, 368.1	Subjective visual disturbances
ICD-9, 368.10	Subjective visual disturbance
ICD-9, 368.13	Asthenopia, eye strain, photophobia

**Table 2:** Experimental independent variables, their levels, and measurement units.

Factor	Levels	Measurements
Alignment errors	Vertical shift	0.5′
	Rotational errors	0.25′
	Magnification differences	6.25%

**Table 3:** Descriptive statistics for hemodynamic responses.

Alignment errors	Statistic	*l* DLPFC		*r* DLPFC	
		Hbb	HbO_2_	Hbb	HbO_2_
Vertical shift	*H*_max_	1.199	0.955	1.054	1.597
	*H*_mean_	−0.29	−0.625	−0.25	−0.308
	*H*_stdev_	0.666	0.608	0.717	0.781
Rotational error	*H*_max_	0.916	1.199	1.43	1.561
	*H*_mean_	−0.204	−0.523	0.068	−0.665
	*H*_stdev_	0.766	0.656	0.857	0.873
Magnification difference	*H*_max_	0.914	0.928	0.855	1.514
	*H*_mean_	0.276	−0.563	0.151	−0.413
	*H*_stdev_	0.641	0.657	0.71	0.764

**Table 4:** Pearson correlation coefficient using hemodynamic response variable.

	*l* DLPFC-Hbb	*l* DLPFC-HbO_2_	*r* DLPFC-Hbb	*r* DLPFC-HbO_2_
*l* DLPFC-Hbb	1			
*l* DLPFC-HbO_2_	0.37	1		
*r* DLPFC-Hbb	0.55	0.39	1	
*r* DLPFC-HbO_2_	−0.43	0.44	0.64	1

**Table 5:** ANOVA summary of significant effect of hemodynamic responses for the alignment errors using transformed data.

Location	Hemodynamic responses	Tukey post ad hoc analyses for alignment display errors (*P* < 0.05)
*r* DLPFC	Deoxygenated hemoglobin (Hbb)	(Magnification difference and rotation error) and (magnification difference and vertical shift)
*l* DLPFC	Oxygenated Hemoglobin (HbO_2_)	(Magnification difference and rotation error) and (magnification difference and vertical shift)
	Oxygenation (Hbb)	(Magnification difference and rotation error) and (magnification difference and vertical shift)
